# PEG-Chitosan Hydrogel with Tunable Stiffness for Study of Drug Response of Breast Cancer Cells

**DOI:** 10.3390/polym8040112

**Published:** 2016-03-26

**Authors:** Fei-Chien Chang, Ching-Ting Tsao, Anqi Lin, Mengying Zhang, Sheeny Lan Levengood, Miqin Zhang

**Affiliations:** 1 Department of Materials Science and Engineering, University of Washington, 302L Roberts Hall, Seattle, WA 98195, USA; feicc@uw.edu (F.-C.C.); chingting.tsao@gmail.com (C.-T.T.); lebject@uw.edu (A.L.); sklanlev@gmail.com (S.L.L.); 2 Department of Molecular Engineering and Science Institute, University of Washington, Seattle, WA 98195, USA; myz16@uw.edu

**Keywords:** hydrogel, chitosan, stiffness, modulus, tunable, extracellular matrix

## Abstract

Mechanical properties of the extracellular matrix have a profound effect on the behavior of anchorage-dependent cells. However, the mechanisms that define the effects of matrix stiffness on cell behavior remains unclear. Therefore, the development and fabrication of synthetic matrices with well-defined stiffness is invaluable for studying the interactions of cells with their biophysical microenvironment *in vitro*. We demonstrate a methoxypolyethylene glycol (mPEG)-modified chitosan hydrogel network where hydrogel stiffness can be easily modulated under physiological conditions by adjusting the degree of mPEG grafting onto chitosan (PEGylation). We show that the storage modulus of the hydrogel increases as PEGylation decreases and the gels exhibit instant self-recovery after deformation. Breast cancer cells cultured on the stiffest hydrogels adopt a more malignant phenotype with increased resistance to doxorubicin as compared with cells cultured on tissue culture polystyrene or Matrigel. This work demonstrates the utility of mPEG-modified chitosan hydrogel, with tunable mechanical properties, as an improved replacement of conventional culture system for *in vitro* characterization of breast cancer cell phenotype and evaluation of cancer therapies.

## 1. Introduction

Tissue extracellular matrix (ECM) provides mechanical support for cells and constitutes an array of biochemical and biophysical factors that influence cellular behavior and fate [1]. Many recent studies focus on understanding the influence of ECM biochemical cues on cell behavior [2,3,4,5]. It is known that biophysical factors associated with the microenvironment are critical to cell fate [6,7,8]. One such biophysical factor, ECM stiffness, modulates cell behavior as anchorage-dependent cells sense and respond to variations in stiffness [1,9,10,11,12]. Importantly, it has long been hypothesized that changes in ECM stiffness are linked to malignant cell phenotype [13,14,15]. For example, increased ECM stiffness and density were reported as directly predictive of breast cancer risk [14]. Many studies linking matrix stiffness with cell malignancy utilize synthetic hydrogel substrates with controllable stiffness for *in vitro* cell culture.

The development of synthetic matrices for *in vitro* studies of cell response to ECM stiffness has proven challenging [16]. A variety of materials, both synthetic and naturally occurring, are utilized to fabricate matrices of varying stiffness and used for *in vitro* cell culture including polyethylene glycol [17], RGD-modified agarose or alginate [18], collagen-coated polyacrylamide [19], collagen-coated agarose [16], and hyaluronic acid [20]. Within these systems, altering matrix stiffness is predominantly achieved by changing degree of crosslinking via irradiation or use of chemical agents, or by varying hydrogel concentration. For example, reconstituted collagen is widely-utilized for this purpose but the crosslinking agent used to increase matrix stiffness, may itself influence cell behaviors such as migration and proliferation [21]. Altering hydrogel concentration or crosslinking ratio can result in significantly different concentrations of cell-adhesion sites thus mediating cell behavior in a way that is not solely matrix stiffness-dependent [13]. In addition, polyacrylamide gels do not possess desirable properties in terms of biodegradation, and hyaluronic acid is expensive. Thus cost, biodegradability, and cytotoxicity continue to pose significant experimental limitations.

**Figure 1:**
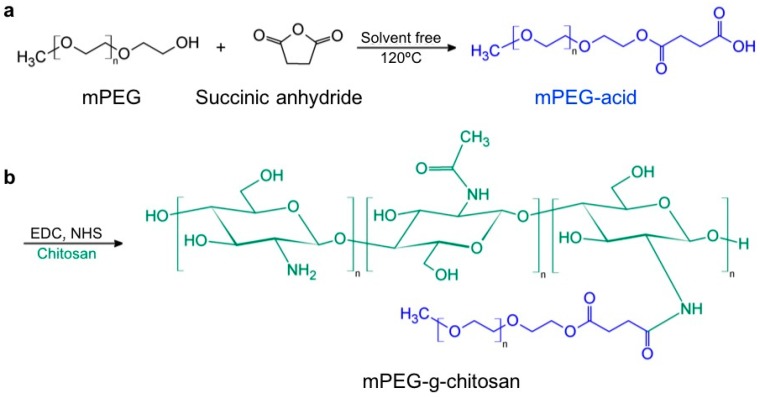
Synthetic route and chemical structure of (**a**) mPEG-acid and (**b**) mPEG-*g*-chitosan.

To circumvent these limitations, we developed a simple, inexpensive hydrogel-based platform composed of methoxypolyethylene glycol grafted chitosan (mPEG-*g*-chitosan) (Figure 1). PEG is one of a limited number of biocompatible, synthetic polymers approved by the U.S. Food and Drug Administration (FDA) for biomedical applications [22,23], whereas chitosan is a natural, biodegradable polysaccharide derived by the partial deacetylation of chitin. Importantly, chitosan shares structural similarities to the glycosaminoglycans (GAG) present in native ECM [24,25,26]. mPEG-*g*-chitosan undergoes sol-gel transition in aqueous solutions at neutral pH and at physiologically relevant temperatures. Hydrogel stiffness is tuned independently of hydrogel concentration by varying grafting efficiency (or PEGylation) in a controlled manner. Varying grafting efficiency varies the interplay of non-covalent/hydrophobic interactions associated with chitosan polymer blocks and hydrophilic interactions associated with the presence of PEG that define overall hydrogel properties [24,27,28]. mPEG-*g*-chitosan hydrogels were characterized via rheological analysis to thoroughly evaluate the effect of PEGylation on gelation temperature and mechanical properties. The hydrogel was then utilized as a platform to investigate stiffness-mediated responses of mouse breast cancer cells in terms of cell morphology, proliferation and drug resistance.

## 2. Materials and Methods

### 2.1. Mateirals

All chemicals were purchased from Sigma-Aldrich (St. Louis, MO, USA) unless otherwise specified. Chitosan (75%–85% deacetylated, medium molecular weight), methoxypolyethylene glycol (mPEG, 750 Da), succinic anhydride, 1-ethyl-3-(3-dimethylaminopropyl) carbodiimide (EDC), *N*-hydroxysuccinimide (NHS), and phosphate buffered saline (PBS) were used as received. Diethyl ether was purchased from J.T. Baker Chemical Company (Phillipsburg, NJ, USA).

### 2.2. Synthesis of Carboxylic Acid-Terminated Methoxypolyethylene Glycol (mPEG-Acid)

Carboxylic acid-terminated methoxypolyethylene glycol (mPEG-acid) was prepared with slight modifications to previously reported methods [22,23]. Briefly, mPEG was dehydrated at 50 °C under vacuum for 8 h before initiating the reaction. Succinic anhydride was added to dehydrated mPEG, and the mixture heated at 100 °C for 30 min to allow the succinic anhydride to dissolve. The molar ratio of succinic anhydride to mPEG was 1.8. The reaction was performed at 120 °C for 8 h, with condenser and circulating cooling water applied. During the reaction, some succinic anhydride condensed inside the reaction vessel. Therefore, excess succinic anhydride was used to ensure complete reaction of the mPEG terminal hydroxyl groups. Diethyl ether was used for extraction after the reaction. Carboxylic mPEG-acid was collected from the organic layer and diethyl ether was removed via vacuum. The yield was approximately 50% by weight. The mPEG-acid was stored at −20 °C for future use.

### 2.3. Synthesis of Methoxypolyethylene Glycol-g-Chitosan (mPEG-g-Chitosan)

As indicated in Table 1, a specific amount of chitosan was dissolved in 30 mL of acetic acid (0.33% *v*/*v*). mPEG-acid was added and the two components mixed with constant stirring until homogeneous. A catalyst solution was prepared by adding EDC (0.2 g) and NHS (0.12 g) in 20 mL of DI water. Subsequently, the catalyst solution was added dropwise into the mixture of chitosan and mPEG-acid. The solution was stirred for 4 h at room temperature allowing for amide linkage formation between chitosan and mPEG-acid [23,29,30]. An amount of 0.5 M NaOH was added dropwise until a pH value of 7 was reached. The solution was dialyzed (molecular weight cutoff 12,000–14,000 Da) three times against DI water to ensure removal of unreacted chemicals and salts. The solution was frozen with liquid nitrogen and mPEG-*g*-chitosan was obtained using lyophilization at −89 °C. The yield was approximately 60%.

Dry mPEG-g-chitosan was reconstituted to make mPEG-g-chitosan hydrogel solutions. As an example of the nomenclature used herein, mPEG-g13-chitosan indicates a grafting efficiency of 13% (molar ratio) as determined by ^1^H NMR.

### 2.4. ^1^H Nuclear Magnetic Resonance Spectroscopy (^1^H NMR) Analysis

The chemical structures of reactants and products were confirmed using ^1^H nuclear magnetic resonance spectroscopy (^1^H NMR, Bruker AV-500, Bruker BioSpin, Rheinstetten, Germany) with the spectra acquired at 500 MHz. The spectra of mPEG-acid, mPEG and succinic anhydride (10–20 mg) were acquired at 25 °C with samples dissolved in DMSO-d_6_ (0.7 mL). mPEG-g-chitosan samples (3–5 mg) were dissolved in D_2_O (0.7 mL) with the addition of one drop of acetic acid-d_4_. The chemical structure and the degree of PEG-grafting (PEGylation) of mPEG-*g*-chitosan were confirmed at 50 °C. PEGylation was defined by the molar ratio of H1 to H7 using the integral function in Topspin (Bruker, Billerica, MA, USA) [25].

### 2.5. Rheological Analysis

The viscoelastic properties of mPEG-*g*-chitosan were characterized by rheological analysis. Specifically, water-soluble mPEG-*g*-chitosan was reconstituted with 1X PBS to yield 1% *w/v* solutions. The solutions were maintained on ice for 4 h with periodic vortexing to ensure full dissolution. Samples were measured using a stress-controlled rheometer (MCR 301, Anton Paar, Ostfildern, Germany) with a cone and plate configuration of 24.982 mm diameter and 0.994° cone angle. A layer of light mineral oil was carefully applied to prevent water evaporation during the experiment.

Measurements of thermally-induced gelation were taken in a dynamic oscillatory mode with a constant frequency of 1 Hz and 1%–5% (no effect on gel formation) strain with temperature ramping at a rate of 1 °C/min. The values of storage and loss modulus (*G*’ and *G*”, respectively) and phase angle (Θ) were obtained accordingly. The incipient of gel network formation, which is defined by the gelation temperature, is given by the crossover of *G*’ and *G*” [31]. The measurement of the gelation temperature showed a good reproducibility. To characterize gel recovery (polymer network restoration after 1000% strain applied for 180 s), viscosity and storage modules (*G*’) were monitored at constant frequency of 1 Hz with 5% strain (no effect on gel formation) as a function of time.

### 2.6. Scanning Electron Microscopy (SEM) Evaluation of Polymer Morphology

Lyophilized samples were placed on double-sided tape, sputter coated with Au/Pd for 70 s at 18 mA and observed with a scanning electron microscope (Model JSM-5600, JEOL Technics, Tokyo, Japan) at an operating voltage of 5 kV, spot size 3.

### 2.7. Cell Proliferation Analysis

Dulbecco’s modified Eagle media (DMEM), Dulbecco’s phosphate-buffered saline (DPBS) and growth factor-reduced Matrigel™ were purchased from Corning (Tewksbury, MA, USA). Mouse mammary carcinoma (MMC) cells were obtained from spontaneous tumors formed in neu-transgenic (neu-tg) mice (FVB/N-TgN(MMTVneu)-202Mul, Charles River Laboratory, Bar Harbor, ME, USA) and transfected with pRFP-N2 as previously reported [24].

MMC cell proliferation was evaluated on 2D tissue culture polystyrene (TCPS), Matrigel™ and mPEG-g-chitosan of varying grafting densities. mPEG-g-chitosan hydrogel was obtained by reconstitution of dried mPEG-*g*-chitosan with DMEM. The solution was maintained on ice for 4 h with periodic vortexing to ensure complete dissolution. Matrigel was thawed at 4 °C overnight. Pre-chilled pipet tips and 48-well tissue culture plates were used for coating. A volume of 100 μL of Matrigel and mPEG-*g*-chitosan hydrogel were pipetted into the wells, respectively, which were then heated to 37 °C for 2 h to induce gelation. MMC cells (10^4^) in 20 μL fully supplemented DMEM were seeded into uncoated tissue culture polystyrene (TCPS), Matrigel-precoated, and mPEG-*g*-chitosan-hydrogel-precoated wells. Fully supplemented DMEM medium was added 1 h after seeding. MMC cell proliferation was measured with Alamar Blue (Sigma Aldrich, St. Louis, MO, USA) after one, three and five days in culture. Briefly, media were gently aspirated and replaced with Alamar Blue working reagent (10× dilution with DMEM, 110 μ g/mL resazurin). After 2 h at 37 °C, the Alamar Blue solution was collected and transferred to a 96-well black bottomed plate. The fluorescence intensity of the solution was measured (560 nm excitation and 590 nm emission) using a SpectraMax M2 microplate reader (Molecular Device, Sunnyvale, CA, USA). The cell number was determined from calibration curves generated with known numbers of MMC cells. MMC cellular aggregates were imaged at the indicated time points using a Nikon TE300 (Nikon, Tokyo, Japan) inverted microscope.

### 2.8. Dose-Dependent Cytotoxicity Analysis

MMC cells (6 × 10^3^) were seeded in uncoated TCPS, Matrigel pre-coated, and mPEG-*g*-chitosan-hydrogel pre-coated 48-well plates. After 2 days of culture, cells were exposed to fully supplemented cell culture media containing doxorubicin (DOX) at various concentration (0, 0.01, 0.1 and 1 μg/mL). The cell viability was assessed with Alamar Blue after 48 h. Cell viability is reported as the percentage of viable cells relative to that of untreated controls (DOX 0 μg/mL).

### 2.9. Statistical Analysis

The results are presented as mean values ± standard deviation (*n* ≥ 3). The significant difference was evaluated by unpaired two-sample Student’s *t*-test. The difference was considered statistically significant when *p* < 0.05 (*).

## 3. Results and Discussion

### 3.1. Chemical Structure of mPEG-g-Chitosan

The mPEG-*g*-chitosan synthetic route shown in Figure 1 involves a two-step reaction: (1) ring opening reaction of succinic anhydride with mPEG to form mPEG-acid (Figure 1a); and (2) amide linkage formation between mPEG-acid and chitosan under EDC/NHS catalysis at room temperature (Figure 1b). The relative proportions of chitosan, mPEG-acid, EDC, and NHS are summarized in Table 1, with samples specified by the PEGylation (%) as determined post-synthesis by ^1^H NMR spectroscopy.

**Figure 2:**
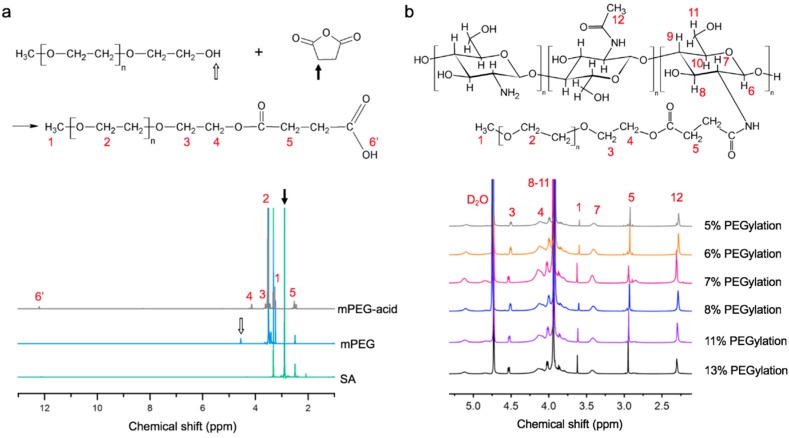
Chemical structures and ^1^H NMR spectra of (**a**) mPEG-acid (gray), mPEG (blue), succinic anhydride (SA, green) and (**b**) mPEG-*g*-chitosan prepared under different conditions resulting in different PEGylations.

The chemical structures of succinic anhydride (SA), mPEG and mPEG-acid were analyzed with ^1^H NMR spectroscopy (Figure 2a). The characteristic peak of SA was assigned at 2.8–2.9 ppm (black arrow, Figure 2a). Characteristic peaks of mPEG located at approximately 3.7 and 4.5 ppm (open arrow, Figure 2a) represent protons on the methylene bridges and hydroxyl protons, respectively. The spectrum of mPEG-acid includes characteristic peaks H4, H5 and H6’. H6’ represents a carboxylic acid group, previously reported in the range of 11.9–12.5 ppm [32,33]. The H6’ peak at approximately 12 ppm in the mPEG-acid spectrum indicates that carboxylic acid was substituted onto mPEG. Compared to the mPEG spectrum, mPEG-acid shows neither characteristic peaks of mPEG at 4.5–4.6 ppm nor SA at 2.8–2.9 ppm. Thus, the final product of mPEG-acid did not contain significant amounts of unreacted mPEG or residual SA thereby confirming its chemical structure and purity.

The chemical structures of mPEG-*g*-chitosan of varying PEGylation were also confirmed by ^1^H NMR analysis (Figure 2b). mPEG was confirmed by the intense signal of H1 on MeO (CH_3_–O–) at 3.6–3.7 ppm [34]. The linkage between mPEG and chitosan was confirmed by the appearance of the peak at 2.9–3.0 ppm (H5), indicating a newly formed amide bond (–NH–CO–CH_2_CH_2_–) as compared to pure chitosan [35]. The grafting efficiency of mPEG onto chitosan was defined by the molar ratio of H1 (the methylene group on mPEG) to H7 (single-bonded hydrogen on the chitosan backbone) as shown in Figure 2b. The ratio of H1 to H7 was determined using the integral function in Topspin (Bruker, Billerica, MA, USA). The degree of chitosan PEGylation was higher when the mPEG-*g*-chitosan was synthesized with a higher mPEG-to-chitosan ratio (Table 1). mPEG (0.43 g) reacted with 0.25, 0.3, 0.35, 0.4, 0.45, and 0.5 g chitosan resulted in grafting efficiencies of 5–8, 11, and 13% (mol/mol), respectively.

### 3.2. Rheological Properties and Stiffness of mPEG-g-Chitosan Hydrogels

Rheological properties, including storage and loss moduli, thermoresponsive gelation and hydrogel restoration of mPEG-*g*-chitosan were measured over a range of PEGylations, to fully characterize the hydrogels and understand the effect of PEGylation on resultant hydrogel properties.

#### 3.2.1. Thermally-Induced Gelation

**Figure 3:**
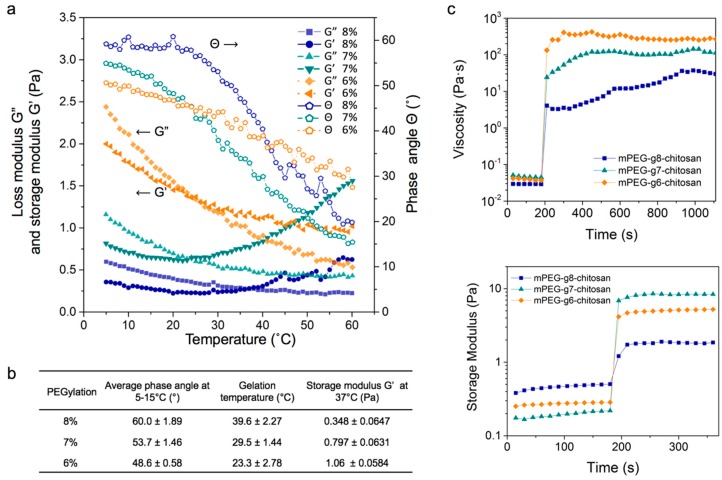
The rheological properties of mPEG-*g*-chitosan (6%, 7% and 8% of PEGylation). (**a**) Phase angle, storage and loss moduli as a function of temperature for 1% *w/v* hydrogel in 1X PBS; (**b**) Summary of gelation temperature, storage modulus, and the average phase angle over 5–15 °C; (**c**) Hydrogel restoration behavior assessed by viscosity and storage modulus, respectively.

Figure 3a shows the thermoresponsive gelation behavior of mPEG-*g*-chitosan reconstituted in 1X PBS at 1% *w*/*v*. As the temperature increased, the loss modulus (*G*”) decreased and crossed over storage modulus (*G*’) at a certain point. This occurred at a phase angle of Θ = 45° and defines the sol-gel transition temperature [36]. The gelation temperature decreased from 37 to 25 °C with decreasing PEGylation. mPEG-*g*5-chitosan was difficult to dissolve in 1X PBS and the gelation temperatures of mPEG-*g*11-chitosan and mPEG-*g*13-chitosan were higher than 37 °C (data not shown). Those three conditions were not useful for *in vitro* cell culture studies, and therefore subsequent rheological analyses and cell culture experiments focused on mPEG-*g*6-chitosan, mPEG-*g*7-chitosan, and mPEG-*g*8-chitosan solutions and hydrogels.

#### 3.2.2. Hydrogel Mechanical Properties

In comparing the behavior of mPEG-*g*-chitosan of different PEGylations, both storage modulus (*G*’) and loss modulus (*G*”) at 37 °C increased with decreasing PEGylation (Figure 3a). The storage modulus, a property correlated with elastic modulus/stiffness, was measured at 37 °C as 0.348, 0.797 and 1.06 Pa for mPEG-*g*8-chitosan, mPEG-*g*7-chitosan, and mPEG-*g*6-chitosan, respectively, indicating an increase in hydrogel stiffness with decreasing PEGylation (Figure 3b). As PEGylation decreased, the average phase angle at 5–15 °C decreased from 60.0 to 48.6 °C, indicating an increasing elastic portion of the viscoelastic behavior. Thus, by controlling only PEGylation of mPEG-*g*-chitosan hydrogels, the storage modulus can be manipulated and optimized base on the intended application.

To understand the role of PEGylation on mPEG-*g*-chitosan properties, it is important to consider the mechanisms that govern the sol-gel transition. Pure chitosan dissolves under acidic conditions because amine groups are protonated and chitosan chains repel due to electrostatic interactions. Under neutral, aqueous conditions, important to the study of biological systems, a gel-like precipitate forms. This is due to neutralization of chitosan amine groups leading to extensive hydrogen bonding and hydrophobic interactions between chains and thus, chain entanglement [37]. mPEG-*g*-chitosan maintains a homogenous solution state at neutral pH and lower temperatures because hydrogen bonding between grafted mPEG and water molecules dominates. Upon heating toward the gelation temperature, the mobility of polymer chains increases. The mPEG follows an inherent tendency towards dehydration [38,39] the hydrogen bonds weaken and strong interactions between water and both chitosan and mPEG are lost. Hydrophobic interactions among chitosan chains prevail above the gelation temperature, creating physical junction zones of polymer chain segments [39].

As PEGylation decreases, there are fewer hydrophilic mPEG moieties present to hinder hydrophobic interactions. The increase in the contribution of hydrophobic interactions among chitosan chains promotes gel network formation and the onset of gelation occurs at lower temperatures compared to the samples with high PEGylations. Correspondingly, the hydrogel storage modulus at 37 °C increases when PEGylation decreases.

#### 3.2.3. Hydrogel Restoration

In Figure 3c, viscosity and storage modulus were recorded over a time sweep during and directly following the cessation of a 1000 s^−1^ strain rate and 1000% strain for 180 s, respectively, because maintenance of integrity following deformation is a fundamental property of hydrogels. When subjected to high shear rate and high strain deformation, the gel behaved as a liquid with a decrease in viscosity and storage modulus. The gel state recovered from deformation rapidly upon lowering the shear rate from 1000 to 0.314 s^−1^ (viscosity measurement) and the strain from 1000% to 5% (storage modulus measurement). All mPEG-*g*-chitosan hydrogels displayed extraordinary resistance to high deformation due to fast self-assembly processes that restore the gel network immediately following removal of the applied strain [40].

### 3.3. Morphology of Lyophilized mPEG-g-Chitosan

**Figure 4:**
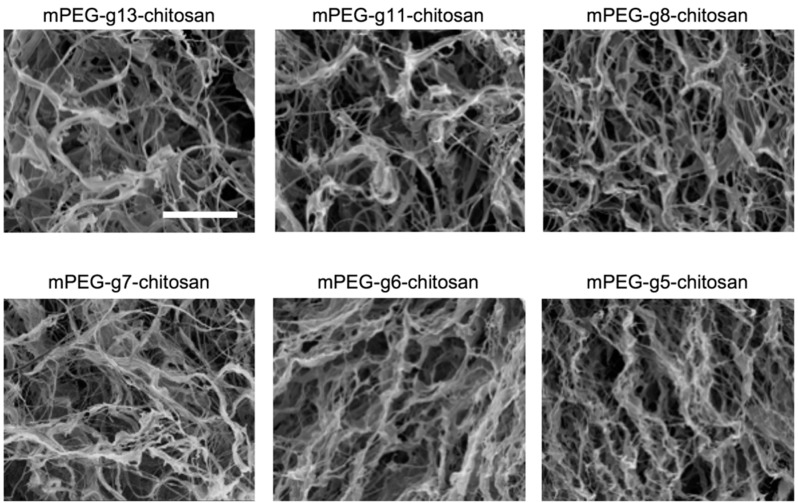
Scanning electron micrographs of lyophilized mPEG-*g*-chitosan representing varying grafting efficiencies (PEGylations). The scale bar represents 20 µm.

The morphology of lyophilized mPEG-*g*-chitosan was observed using SEM to examine the architecture and pore structure. The lyophilization process resulted in dry mPEG-*g*-chitosan with similar morphology/porosity and no microscale phase separations regardless of degree of PEGylation (Figure 4). Therefore, the rheological properties of hydrogels of varying PEGylation are dominated by the chemical structure and not by mPEG-*g*-chitosan architecture created during the drying process.

### 3.4. MMC Cell Behavior on mPEG-g-Chitosan Hydrogels of Varying Stiffness

Breast cancer tumors, unlike normal breast tissue, are heterogeneous in terms of mechanical properties with higher stiffness at their periphery [41,42]. *In vitro* studies show that matrix stiffness influences cancer cell behavior in both 2D and 3D culture environments [24,43,44,45,46]. Taken together, these results indicate the importance of developing materials to further investigate the role of tissue stiffness in the tumor microenvironment. Here, mPEG-g-chitosan hydrogels were utilized as a synthetic ECM to evaluate the biophysical response of MMC cells to matrices of varying stiffness in terms of cell proliferation, morphology, and drug resistance.

#### 3.4.1. MMC Cell Proliferation and Morphology

**Figure 5:**
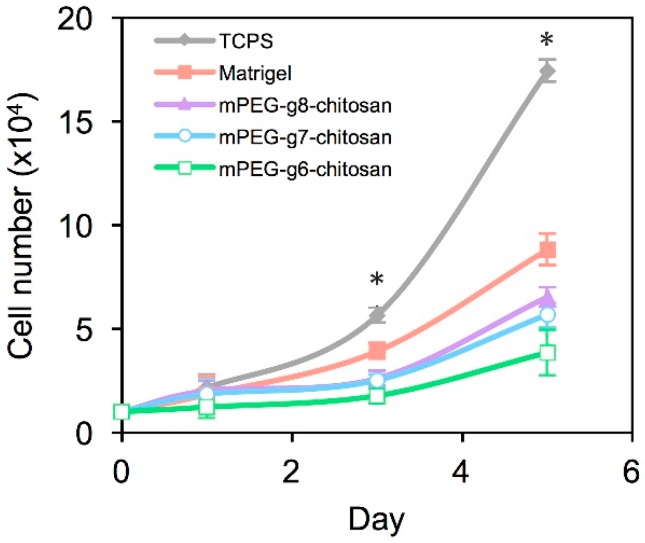
Effect of culture environment on MMC cell proliferation. Results are presented as mean ± standard deviation and * indicates statistically significant differences at the given time points (*p* < 0.05).

The proliferation rate and morphology of MMC cells was compared among cultures on 2D TCPS, Matrigel, and mPEG-*g*-chitosan hydrogels with 6%, 7%, and 8% PEGylation. Figure 5 shows that MMC cell proliferation rate was lower for cultures on Matrigel and mPEG-*g*-chitosan hydrogels as compared with cultures on TCPS. On day 3, the number of cells on TCPS was greater than all other conditions and the number of cells on Matrigel was greater than all mPEG-*g*-chitosan conditions (*p* < 0.05). On day 5, the number of cells on mPEG-*g*6-chiotsan hydrogel was lower (*p* < 0.05) than the cell number on mPEG-*g*7-chiotsan and mPEG-*g*8-chitosan, which have lower storage modulus/stiffness, and significantly lower than the number of cells on Matrigel (*p* < 0.05) and TCPS (*p* < 0.05). The difference in proliferation rates may be attributed to accessibility to bulk media as well as increased cell-cell and cell-ECM interactions associated with the hydrogel conditions, which represent a 3D environment as cells can invade the matrices. Cells on TCPS were exposed uniformly to bulk media with limited cell-cell interactions associated with monolayer culture. Matrigel and mPEG-*g*-chitosan gels likely enhance cell-matrix adhesion, cell-cell interactions, thereby delaying proliferation as cells acclimate to the new environment. In addition, the hydrogel may facilitate the development of nutrient and biochemical gradients, thereby affecting proliferation [47].

**Figure 6:**
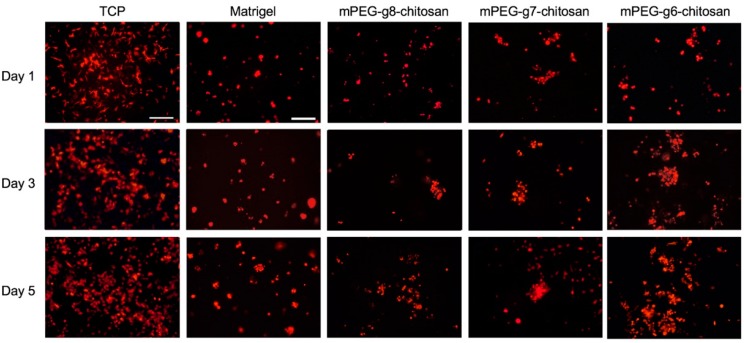
Effect of culture environments on MMC cell morphology and organization. Fluorescence imaging of MMC cells cultured on TCP, Matrigel, and mPEG-*g*-chitosan hydrogels of different PEGylation for 1, 3, and 5 days. The scale bar represents 200 µm.

Figure 6 confirms that cells formed a monolayer on TCPS and exhibited a polarized, elongated morphology, whereas cultures on Matrigel and mPEG-*g*-chitosan hydrogels resulted in formation of multicellular aggregates. Multicellular spheroids were present on Matrigel while the aggregates on mPEG-*g*-chitosan hydrogels exhibited irregular shapes. The cell clusters on the stiffest gel, mPEG-*g*6-chitosan, became larger over time. The cell clusters on softer hydrogels did not increase in size as significantly, but more clusters appear over time. These results suggested that on stiffer gels, where proliferation is relatively slower, cell–cell interactions and likely cell migration promoted aggregation [48]. These results also correspond to other studies demonstrating that increased ECM stiffness corresponds with malignant phenotype in mammary epithelium [13], the perturbation of epithelial morphogenesis [46], and cell colony size increased with stiffening matrix [46,49].

#### 3.4.2. MMC Cell Response to Doxorubicin

**Figure 7:**
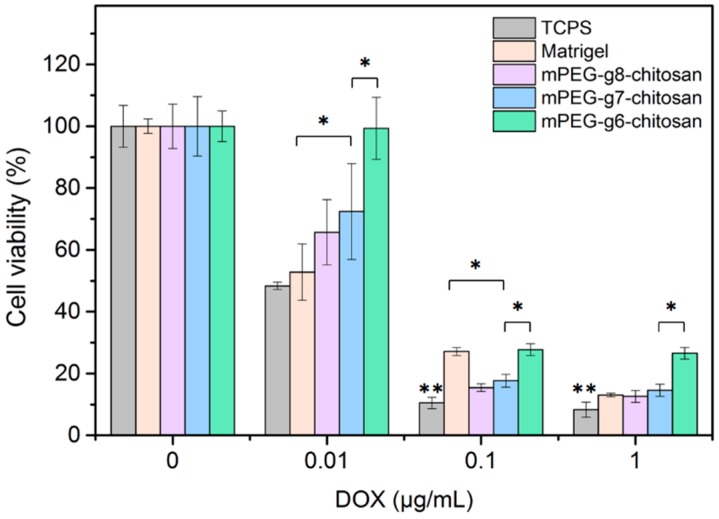
Assessment of MMC cell drug response when cultured on TCPS, Matrigel, and mPEG-*g*-chitosan hydrogels with 6%, 7%, and 8% PEGylation. Results are presented as mean ± standard deviation and * indicates significant difference (*p* < 0.05); ** indicates significant difference compared with all other conditions (*p* < 0.05).

Doxorubicin (DOX) is a commonly used chemotherapy agent for treating multiple cancers including breast tumors. MMC cells were seeded on TCPS, Matrigel and mPEG-g-chitosan hydrogels, allowed to acclimate for 48 h and then exposed to DOX at 0, 0.001, 0.01, and 1 μg/mL. Cell viability was assessed after 48 h. As shown in Figure 7, overall cell viability was significantly higher for cells cultured on Matrigel or mPEG-*g*-chitosan hydrogels than cells cultured on TCPS. This result is similar to the results from previous studies on human breast cancer cell line T4-2 [50], ovarian cancer cells [43], and prostate cancer cells [51]. Among all hydrogel conditions tested, MMC cells cultured on the stiffest hydrogel, mPEG-*g*6-chitosan, was found to attenuate the cytotoxic effects of DOX most significantly, resulting in higher viability post-treatment. This result suggests that the cells grown on the stiffest hydrogel showed enhanced malignancy, which may be partially attributed to enhanced cell-matrix interaction. [47] Our results correlate with studies utilizing other 3D gel systems where cancer cells grown on stiffer gels formed dense cell aggregates and exhibited higher malignancy, perhaps resulting from elevated integrin signaling, Rho activity, increased focal adhesion formation and ERK signaling, or up-regulated survival signaling [44,46,48,52,53,54,55].

## 4. Conclusions

We developed a simple, 3D, hydrogel platform with tunable stiffness that does not require alterations in polymer concentration or the use of crosslinking agents to induce changes in stiffness. The gel state of mPEG-*g*-chitosan is easily triggered with elevated temperature under physiologically relevant conditions. The gelation temperature and stiffness are modulated by changing the PEGylation of chitosan polymer chains where decreased PEGylation correlates with increased gel stiffness as measured in terms of storage modulus. Furthermore, the hydrogel demonstrated instant network restoration after decrease of high shear rate and strain, which is an important hallmark of hydrogels. *In vitro* investigation of mouse mammary carcinoma cell response to mPEG-*g*-chitosan matrix showed that cells cultured on the stiffest hydrogel condition attained a more malignant phenotype as evidenced by increased drug resistance. This material represents an important model of tumor ECM for the study of cellular response to biophysical changes in the tumor microenvironment.

## Figures and Tables

**Table 1:** Conditions for the preparation of mPEG-*g*-chitosan with different grafting efficiencies. The nomenclature used herein defines mPEG-*g*13-chitosan as mPEG-*g*-chitosan with a grafting efficiency of 13% (molar ratio) as determined via ^1^H NMR. The number “*n*” following the “*g*” in mPEG-*gn*-chitosan denotes the percent of PEGylation.

Sample	mPEG-acid (g)	Chitosan (g)	EDC (g)	NHS (g)
mPEG-*g*5-Chitosan	0.43	0.50	0.20	0.12
mPEG-*g*6-Chitosan	0.43	0.45	0.20	0.12
mPEG-*g*7-Chitosan	0.43	0.40	0.20	0.12
mPEG-*g*8-Chitosan	0.43	0.35	0.20	0.12
mPEG-*g*11-Chitosan	0.43	0.30	0.20	0.12
mPEG-*g*13-Chitosan	0.43	0.25	0.20	0.12
